# Prognostic Value of Admission Chest CT Findings for Invasive Ventilation Therapy in COVID-19 Pneumonia

**DOI:** 10.3390/diagnostics10121108

**Published:** 2020-12-19

**Authors:** Eva Gresser, Johannes Rueckel, Daniel Puhr-Westerheide, Vincent Schwarze, Nicola Fink, Wolfgang G. Kunz, Dietmar Wassilowsky, Michael Irlbeck, Jens Ricke, Michael Ingrisch, Bastian O. Sabel

**Affiliations:** 1 Department of Radiology, University Hospital, LMU Munich, 81377 Munich, Germany; johannes.rueckel@med.uni-muenchen.de (J.R.); daniel.puhr-westerheide@med.uni-muenchen.de (D.P.-W.); vincent.schwarze@med.uni-muenchen.de (V.S.); nicola.fink@med.uni-muenchen.de (N.F.); Wolfgang.Kunz@med.uni-muenchen.de (W.G.K.); Jens.Ricke@med.uni-muenchen.de (J.R.); michael.ingrisch@med.uni-muenchen.de (M.I.); bastian.sabel@med.uni-muenchen.de (B.O.S.); 2 Department of Anaesthesiology, University Hospital, LMU Munich, 81377 Munich, Germany; Dietmar.Wassilowsky@med.uni-muenchen.de (D.W.); michael.irlbeck@med.uni-muenchen.de (M.I.)

**Keywords:** COVID-19, SARS-CoV-2, diagnostic imaging, intensive care, risk factors

## Abstract

(1) Background: To assess the value of chest CT imaging features of COVID-19 disease upon hospital admission for risk stratification of invasive ventilation (IV) versus no or non-invasive ventilation (non-IV) during hospital stay. (2) Methods: A retrospective single-center study was conducted including all patients admitted during the first three months of the pandemic at our hospital with PCR-confirmed COVID-19 disease and admission chest CT scans (*n* = 69). Using clinical information and CT imaging features, a 10-point ordinal risk score was developed and its diagnostic potential to differentiate a severe (IV-group) from a more moderate course (non-IV-group) of the disease was tested. (3) Results: Frequent imaging findings of COVID-19 pneumonia in both groups were ground glass opacities (91.3%), consolidations (53.6%) and crazy paving patterns (31.9%). Characteristics of later stages such as subpleural bands were observed significantly more often in the IV-group (52.2% versus 26.1%, *p* = 0.032). Using information directly accessible during a radiologist’s reporting, a simple risk score proved to reliably differentiate between IV- and non-IV-groups (AUC: 0.89 (95% CI 0.81–0.96), *p* < 0.001). (4) Conclusions: Information accessible from admission CT scans can effectively and reliably be used in a scoring model to support risk stratification of COVID-19 patients to improve resource and allocation management of hospitals.

## 1. Introduction

In December 2019, a severe acute respiratory syndrome coronavirus 2 (SARS-CoV-2) was confirmed as the cause of a new disease, which was termed coronavirus disease 2019 (COVID-19) by the World Health Organization [1]. COVID-19 has since become a global health crisis overwhelming numerous health care systems [1,2]. Up to this point more than 65 million people have tested positive for SARS-CoV-2 and more than one and a half million people have died from the disease [3]. After a steady decline of cases over the summer months in Germany, numbers have been increasing rapidly towards the cold season and a second disease wave exhausts intensive care capacities [3,4]. According to meta-analyses, around 15–30% of hospitalized patients develop a critical course of the disease requiring intensive care unit (ICU) treatment and invasive ventilation (IV), with an ICU mortality rate of around 40–50% which is higher than usually seen in other viral pneumonia [5,6,7,8].

Typical symptoms of COVID-19 disease can include fever, dry cough, chest pain, dyspnea and gastrointestinal symptoms such as nausea and diarrhea [9] which can also be caused by other viruses such as influenza A and B, and can make it difficult to distinguish between these diseases in the upcoming flu season. Polymerase chain reaction (PCR) testing has shown false negatives in the early stages of COVID-19 disease for some cases and test results may take some time, causing difficulties regarding the management of hospital capacities [10]. In this context, chest computed tomography (CT) scans demonstrated their high diagnostic value in cases with initially negative PCR-testing or pending results, and have since been an effective complementary tool for allocation and triage purposes [11,12,13,14,15,16]. While the role of CT scans in disease management has been strengthened, chest radiographs (CXR) seem to be of little diagnostic value in the early stages, but reliably show signs of acute respiratory distress syndrome in later stages and can be considered useful in determining disease progression [16,17]. Regarding CT imaging, typical findings of COVID-19 pneumonia have been described in several prior studies [18,19,20,21]. It has been shown that the extent of lesions correlates with disease severity of the patients [12,20,22,23]. Furthermore, CT findings at various stages of the disease have been studied and connected with clinical severity [23,24,25,26,27,28,29]. In early stages, typical findings include focal or multifocal ground glass opacities (GGOs) that might come with consolidations and interlobular thickening. In later stages, increasing patchy and reticular opacities, air bronchograms as well as subpleural bands may sometimes be observed [24,27,28]. Pleural or pericardial effusion, lymph node enlargement, bronchial wall thickening as well as mucus plugging might be seen more frequently in severe cases or later stages of the disease and may point to potential superimposed infections [18,19,20,25].

The purpose of this study was to analyze our hospitalized patient cohort of confirmed COVID-19 cases in the first wave of the pandemic in Germany (early March to early April 2020) and to determine risk factors for IV therapy in ICU. We hypothesized that prognostic information on the course of the disease can be obtained from available chest CT scans upon admission of the patient. Among other countries, Germany currently faces a second rapid progression of the COVID-19 pandemic. The insights from this study might support patient allocation and thereby improve scarce resources management of hospitals in different health care systems.

## 2. Materials and Methods

### 2.1. Data Collection

Our retrospective single-center study was approved by the local institutional review board. Between the beginning of March and the beginning of April 2020, 69 consecutive patients (22 females, 47 male) with PCR-confirmed COVID-19 disease and worsening of general or respiratory status were in subsequent need of hospitalization. A chest CT scan on admission was mandatory, as well as a full set of clinical, laboratory and epidemiologic data.

Patients were divided into two groups: (I) severe course of the disease in need of invasive ventilation therapy in ICU (IV), and (II) all other patients with either more moderate clinical disease courses or with a patient will against transfer to intensive care units and invasive actions. Patients of group II were transferred to normal or intermediate care units and were not intubated during the hospital stay (non-IV). They either had no need for oxygen supply or received supportive oxygen therapy according to individual needs with the use of nasal prongs and plain masks up to high flow nasal cannula and NIV-therapy. Patients were followed up until decease or dismissal from hospital. PCR testing was either performed during admission or has been performed previously in an outpatient setting with all of the latter cases undergoing re-confirmation during the hospital stay. Basic demographic data such as age, gender and comorbidities were collected. The latter were categorized within obesity, cardiovascular, respiratory as well as immunologic or endocrinologic disorders. Symptoms of the current disease as well as their time of occurrence and evolvement were retrospectively extracted from our database. Relevant laboratory findings at the time point of admission were collected (namely C-reactive protein (CRP), leucocytes, interleukin-6 (Il-6), lactatdehydrogenase (LDH), D-dimer, internationalized normalized ratio (INR) and partial thromboplastin time (aPTT).

### 2.2. Image Acquisition

CT scans were performed using CT scanners of our emergency department (Somatom AS+, Siemens Healthineers, Forchheim, Germany and GE Optima 660, GE Healthcare, Chicago, IL, USA), either as a native high-resolution scan or a contrast-enhanced pulmonary embolism protocol at end inspiration with the patient in supine position. Image acquisition was modulated between 80–120 kVp with adaptive tube current (mAS). All images were reconstructed with a slice thickness of 1.00 mm or 1.25 mm. Multiplanar reconstruction methods were performed on all images.

### 2.3. Image Interpretation

All images were interpreted by three radiologists each with 7, 3, and 3 years of experience as members of the thoracic imaging group (initials blinded). Previously established findings associated with COVID-19 pneumonia [16,25,27] were reviewed and blinded to all clinical data. In detail, GGOs, consolidations, crazy-paving pattern, subpleural bands, air bronchogram, architectural distortions and emphysema as well as reticulations were documented. Bronchial wall thickening, mucus plugging, intrathoracic lymph node enlargement, pleural effusions, pericardial effusion, cardiomegaly, coronary artery disease, signs of pulmonary arterial hypertension and pulmonary embolism (if present) were additionally reported. Distribution of findings was described as lower or upper lobe predominant or both, peripheral or central predominant or both and as either being focal, multifocal or diffuse (meaning coalescing lesions). The number of lobes involved was documented and for a more comprehensive visual quantitative evaluation of lung involvement, a severity score was recorded for every patient, similarly to previous studies [12,20,22,23,30] by assessing the scope of lesions in each lobe: 0 (no lesion), 1 (affecting < 25% of lobe), 2 (affecting > 25% but < 50%), 3 (affecting 50–75% of lobe) or 4 (affecting > 75% of lobe) with the CT lung involvement score as the sum of the 5 lobes (range from 0–20). CXRs taken within 48 h prior to or after the CT scans were evaluated for indicative findings for a pulmonary involvement in COVID-19 disease.

**Figure 1:**
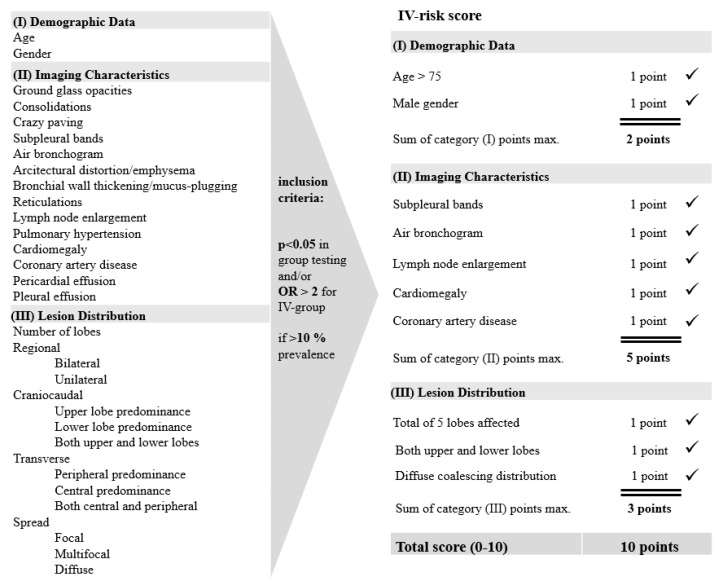
IV-risk score. Flow chart on the development of the IV-risk score with a range from 0–10 assessing the risk for invasive ventilation therapy. All parameters with significant test result (*p <* 0.05) in group testing as well as parameters with odds ratios of >2 were included in the risk score. Parameters were included only if found in >10% of the patient population.

Finally, an empirical risk score for IV-therapy (IV-risk score) was calculated only using parameters that could directly be assessed from the CT scan during standard reporting procedure, ranging from 0–10 with 1 point each for every of the following characteristics in three categories: (I) demographic data (age > 75, male gender), (II) imaging characteristics (subpleural bands, air bronchogram, cardiomegaly, coronary artery disease, lymph node enlargement) and (III) lesion distribution (lesions in both upper and lower lobes, diffuse lesions which coalesce, lesions found in each of the 5 lobes). We included all parameters with a significant test result (*p <* 0.05) between the groups as well as parameters with odds ratios of >2 for high odds of the presence of a given parameter with the invasive ventilation requirement. In order to prevent overinterpretation of effects of only occasionally exhibited parameters, only those parameters present in at least 10% of patient population were included in the score. A flow chart on the development of the IV-risk score with information on inclusion criteria for the used features is displayed in Figure 1.

### 2.4. Statistical Analysis

All statistical analyses were performed with SPSS software (version 26.0, IBM Corp., Armonk, NY, USA). Continuous variables are reported as median (95% confidence interval (CI)) or mean ± standard deviation and Mann-Whitney-U test was applied for testing between groups. Categoric variables were presented as numbers and percentages and compared by chi-square test if applicable or Fisher’s exact test. Significance was defined as a *p*-value < 0.05. Odds ratios were calculated for imaging features. Receiver operating curve (ROC) analysis including areas under the curve served as a measure to quantify the discriminative power of the established scoring system, which aims to predict the necessity of invasive ventilation within the next 21 days. For estimation of the confidence interval of the AUC, calculation was done with R (version 3.5.3) using the pROC package with DeLongs algorithm [31,32].

## 3. Results

### 3.1. Clinical Characteristics and Demographic Data

Twenty-three of the 69 patients (22 men, 1 woman) required invasive ventilation on ICU within the first 21 days after CT acquisition (IV-group). The remaining 46 patients (25 men, 21 women) were hospitalized in normal and intermediate care units (non-IV-group). Median age in the IV-group was 64 years (95% CI 57.5–70.5) versus 61 years (95% CI 56.6–65.4) in the non-IV-group, *p* = 0.944. Male gender turned out to be a significant independent risk factor for IV within our study cohort (96.0% versus 54.0%, *p* < 0.001). Most frequently reported symptoms were fever (81.2%), dry cough (71.0%), reduced general condition (65.2%) and dyspnea (53.6%) followed by gastrointestinal symptoms such as nausea and diarrhea (34.8%). In general, reported symptoms did not substantially differ between IV- and non-IV-patients. While patients in the non-IV-group were admitted on average in their second week of symptoms 1.7 ± 0.73 (mean ± SD), those which turned out to require IV-therapy presented significantly later after onset of symptoms within week number three 2.4 ± 0.75 (mean ± SD), *p* = 0.010. Underlying comorbidities as stated in medical patient reports were similarly distributed between the groups. Patients of the IV-group showed significantly higher values on admission compared to non-IV-group for the following parameters: CRP 11.9 ± 9.9 (mean ± SD) versus 5.5 ± 6.1 (mean ± SD; *p* = 0.004) in mg/dL, Procalcitonin 2.4 ± 5.6 (mean ± SD) versus 0.2 ± 0.5 (mean ± SD; *p* = 0.032) in ng/mL, IL-6 482.3 ± 1351.2 (mean ± SD) versus 34.1 ± 31.6 (mean± SD; *p* < 0.001) in ng/L and LDH 469.6 ± 171.6 (mean ± SD) versus 326.2 ± 209.0 (mean ± SD; *p* < 0.001) in U/L. Coagulation parameters and leucocyte counts were evenly distributed. Average time from admission to intubation was 2.9 ± 3.1 (mean ± SD) days. Hospital stay was significantly longer for the patients in the IV-group (43.6 ± 47.7) compared to the non-IV group (9.7 ± 7.6; *p* < 0.001). In-hospital mortality was significantly higher in the IV-group (21.7% compared to 4.3%; *p* = 0.039). A detailed overview of data is given in Table 1.

### 3.2. Imaging Findings within our Study Cohort

CT evaluations confirmed most typical COVID-19 findings already described in the recent literature, the most predominant findings being ground glass opacities (91.3%), consolidations (53.6%), air bronchogram (46.4%), crazy paving pattern (31.9%) and subpleural bands (34.8%). Most frequent distribution patterns were bilateral (81.2%), peripheral (71.0%) and lower lobe predominant (43.5%) as well as multifocally distributed (60.9%). A substantial number of patients presented with lesions in all five lobes (69.6%). 7.2% of all included patients did not show any signs of pulmonary involvement of confirmed COVID-19 disease at the time of CT imaging. A detailed overview of imaging findings is summarized in Table 2.

### 3.3. Group-related Differences in Imaging Characteristics

**Figure 2:**
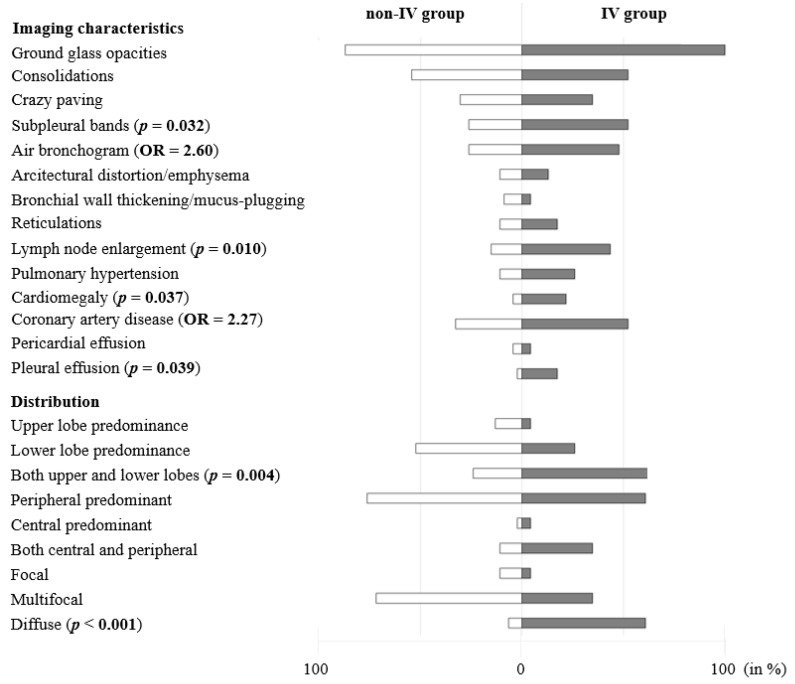
Overview of CT imaging characteristics. Prevalence of imaging characteristics in the invasive ventilation (IV) and non-IV-group in %, corresponding *p*-values (*p*) and odds ratios (OR).

**Figure 3:**
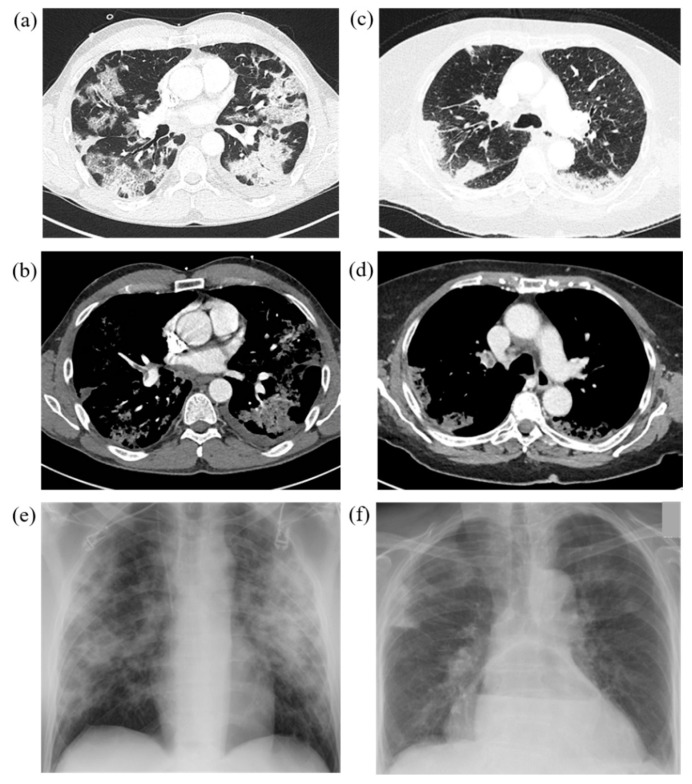
Representative cases with typical COVID-19 findings on chest CT and chest radiograph in both study groups. IV = Invasive ventilation group; non-IV = No/non-invasive ventilation group. (**a**)/(**b**) Chest CT of a 49-year-old, PCR-confirmed male COVID-19 patient in the IV-group after onset of symptoms 15 days ago with immobilization due to reduced general condition and fever, dry cough and headache. CT on admission reveals GGOs, crazy paving pattern, consolidations, subpleural bands in the lower lobes, lymph node enlargements and right central pulmonary embolism. IV risk-score on admission was 7 points out of 10. (**c**)/(**d**) 81-year-old female COVID-19 patient in the non-IV-group with onset of symptoms one week ago presenting with fever, dry cough and reduced general condition, diarrhea and nausea. Central pulmonary embolism in the both pulmonary arteries is noted. GGOs as well as consolidations were observed, partially rated as pulmonary infarction. IV-risk score on admission was 1 point out of 10. Thoracic stomach as secondary finding. (**e**)/(**f**) Corresponding chest radiographs of both cases showed signs of atypical pneumonia as well as pulmonary infarction due to pulmonary embolism.

At baseline CT upon admission, GGOs were found in all patients (100.0%) in need of IV and in most of the patients in the non-IV-group (87.0%), *p* = 0.168. Consolidations (non-IV 54.3%, IV 52.2%, *p* = 0.864), crazy paving pattern (non-IV 30.4%, IV 34.8%, *p* = 0.715), architectural distortion or emphysema (non-IV 28.3%, IV 13.0%, *p* = 0.158) and pericardial effusions (non-IV 4.3%, IV 4.3%, *p* = 1) were found equally. Findings more frequently observed in the IV-group compared to non-IV-group were air bronchogram (43.5% versus 52.2%, *p* = 0.495), reticulations (17.4% versus 10.9%, *p* = 0.448), signs of pulmonary hypertension (26.1% versus 10.9%, *p* = 0.104) and coronary artery disease (52.2% versus 32.6%, *p* = 0.116), with all of these not showing statistical significance. Bronchial wall thickening and mucus plugging were slightly more often seen in the non-IV-group (4.3% versus 8.7%, *p* = 0.551). The presence of subpleural bands in the IV-group was significantly more frequent compared to non-IV-group (52.2% versus 26.1%, *p* = 0.032). Moreover, lymph node enlargement (43.5 versus 15.2, *p* = 0.01), pleural effusions (17.4% versus 2.2%, *p* = 0.039) and cardiomegaly (21.7% versus 4.3%, *p* = 0.037) were noted significantly more often in the IV-group. Contrast-enhanced CT screening for pulmonary embolism was conducted in 39.1% of patients in the IV-group and 15.2% in the non-IV-group and found pulmonary embolism to be present in 11.1% of IV cases and 28.6% of non-IV cases, *p* = 0.550. Chest radiographs taken within 48 h prior or after the CT scan were present in 87% of cases in the IV-group and all showed positive signs of atypical pneumonia (100%), whereas in only 23.9% of patients in the non-IV-group CXR examinations were conducted with 63.6% showing signs of atypical pneumonia. Further details on group differences (IV vs. non-IV) are summarized in Table 2 and Figure 2, a case example is provided in Figure 3.

### 3.4. Group-Related Differences in Distribution Patterns

The number of pulmonary lobes involved proved to be significantly higher in the IV-group with an involvement of 4.9 ± 0.6 (mean ± SD) versus 3.5 ± 1.9 (mean ± SD) in the non-IV-group (*p* = 0.008). The distribution pattern was significantly different between both groups in the craniocaudal dimension with a predominance of lesions in the lower lobes in the non-IV-group and equal distribution of lesions in the upper and lower lobes in the IV-group (*p* = 0.004). No statistically significant difference was found in the transverse dimension with most lesions being located in the lung periphery in both groups. The spread of lesions proved to be significantly different with a predominantly multifocal distribution in the non-IV-group versus a diffuse distribution in the IV-group (*p* < 0.001). Significant difference between both groups was found regarding the CT lung involvement score with 17.52 ± 3.0 (mean ± SD) for IV-group versus 10.76 ± 6.6 (mean ± SD) for non-IV-group, respectively (*p* < 0.001).

### 3.5. IV-Risk Score for Invasive Ventilation Therapy

**Figure 4:**
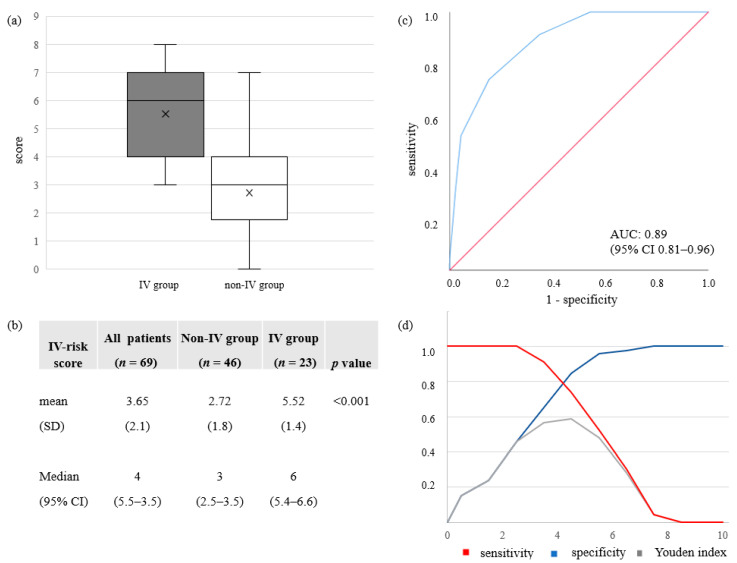
On admission CT IV-risk score analysis. (**a**)/(**b**) Descriptive statistics for the developed IV-risk score for IV-group and non-IV-group with mean (SD) and median (95% CI). (**c**) ROC curve for the diagnostic ability of IV-risk score to differentiate between IV and non-IV-groups on admission CT. In ROC analysis the AUC for the diagnostic probability for IV therapy during hospitalization was 0.89 (95% CI 0.81-0.96). (**d**) Sensitivity, specificity and Youden indices are displayed for different IV-risk scores. Optimal cut-off value based on a maximized Youden index was an IV-risk score of 5 with a sensitivity of 0.81 and a specificity of 0.85. An IV-risk score of 4 resulted in a sensitivity of >0.9 and a specificity of >0.65.

IV-risk scoring using basic demographic and imaging data for the risk stratification of IV therapy proved to be significantly different between both groups with a median of 6 points (95% CI 5.4–6.6) and 5.52 ± 1.4 (mean ± SD) in the IV-group versus a median of 3 points (95% CI 2.5–3.5) and 2.72 ± 1.8 (mean ± SD) in the non-IV-group respectively, *p* < 0.001. ROC analysis showed an area under the curve (AUC) of 0.89 (95% CI 0.81–0.96) for predicting IV-therapy within 21 days of CT scan. ROC operating points have been approximated to the maximum sum of sensitivity and specificity (Youden’s Statistics) and calculated cut-off values with corresponding diagnostic metrics shown in Figure 4. Estimated optimal cut-off value based on a maximized Youden index was an IV-risk score of 5 points with a sensitivity of 0.81 and a specificity of 0.85. For an IV-risk score of 4 points sensitivity was found to be >0.9 with a specificity > 0.65. This section may be divided by subheadings. It provides a concise and precise description of the experimental results, their interpretation as well as the experimental conclusions that can be drawn.

## 4. Discussion

**Figure 5:**
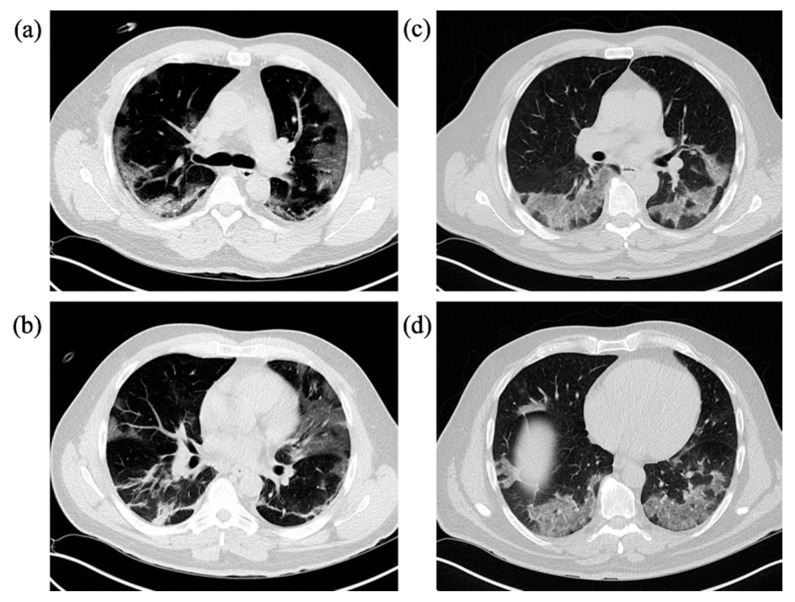
Representative cases with typical COVID-19 imaging features on chest CT in a group comparison. IV = Invasive ventilation group; non-IV = No/non-invasive ventilation group. (**a**)/(**b**) 45-year-old male patient of the IV-group with known contact to a positively tested working colleague. Symptom onset was around 2 weeks earlier and worsened over the last week prior to admission with fever, sore throat and worsening general condition, progressive dyspnea in the preceding four days. IV-risk score of the patient on admission was 5 points with 5 lobes involved. GGOs as well as subpleural bands were the predominant finding in CT scan on admission. (**c**)/(**d**) 59-year-old male patient in the non-IV-group; no known exposure, beginning of symptoms about a week ago with fever, dry cough and reduced general condition. IV-risk score on admission was 2 with 5 lobes involved. GGOs was the predominant finding with some interseptal thickening within the regions, no consolidations.

Early identification of invasive ventilation requirement is a promising strategy for effective resource management during the COVID-19 pandemic. It has already been shown that CT scans are useful in supporting the diagnosis and follow-up of COVID-19 disease and to evaluate the extent of acute respiratory lung involvement [16]. The typical signs of COVID-19 pneumonia in different stages of the disease have been described [20,23,24,25] and linked to the extent of the disease [26,27,28,29]. In our study, critically ill patients in the IV-group exhibited more imaging findings associated with later stages of the disease and superimposed findings: subpleural band-like consolidations, which were seen predominantly in dorsal lower lobes, were significantly more prevalent in the IV-group (IV 52.2% versus non-IV 26.1%, *p* = 0.032), an example is illustrated in Figure 5. They are assumed to show a transformation of GGOs into linear consolidations as a possible sign towards an organizing pneumonia in response to lung injury [19,23,33] and were described in earlier COVID-19 studies at later stages of the disease with a prevalence of up to 28% [20,23,27,28]. We also assume that patients already exhibiting subpleural bands upon hospital admission might either be suffering from an on average more rapid progression of the disease or might have been seeking medical assistance rather late after first symptoms onset compared to patients with a more moderate course of the disease. A late start of treatment in patients with the risk of developing critical impairments might negatively impact the disease course and outcome. Pleural effusion (17.4% versus 2.2%, *p* = 0.039) and lymph node enlargement (43.5% versus 15.2%, *p* = 0.010) were significantly more predominant in the IV-group potentially reflecting signs of systemic inflammation and bacterial superinfection [26,27,29]. This might be supported by the significant difference in elevation of inflammatory laboratory parameters such as Procalcitonin as well as LDH between the groups. Cardiomegaly (21.7% versus 4.3%) was found to be significantly more frequent (*p* = 0.037) in the IV-group and coronary artery disease was observed more often in critically ill patients (52.2% versus 32.6%, *p* = 0.116), presumably reflecting the influence of underlying cardiovascular diseases. The number of lobes affected by lesions was higher for the IV-group (*p* = 0.008) and increasing involvement of both lower and upper lower lobes being observed (*p* = 0.004) with a more diffuse distribution pattern of lesions (*p* < 0.001).

Mortality was significantly higher in the IV-group compared to the non-IV group (21.7% versus 4.3%, *p* = 0.039) and length of stay was significantly longer in the group of IV-patients (43.6 days versus 9.7 days, *p* < 0.001), which stresses the importance for adequate risk assessment for an early and optimal patient care and effective resource management. Risk stratification on admission to hospital is challenged by often incomplete information about the patient and by the immense complexity when it comes to treatment decisions in an emergency setting. Some publications have already discussed how laboratory findings as well as the clinical presentation of patients with confirmed COVID-19 diagnosis might contribute to an adequate risk stratification and to guide treatment decisions [34,35]. Usually, radiological risk assessment is based on the extent of lung involvement. Several previous studies have established scores which take into account the visual quantitative involvement of lung parenchyma to reflect clinical conditions [12,20,22,30]. The extent of lung involvement has been shown to significantly correlate with the severity of the disease. We have determined the quantitative involvement for each patient on admission to hospital in our study cohort and confirmed the positive correlation with the severity of the disease (*p* < 0.001). However, this score is not easily applicable in clinical routine and does not take into account qualitative findings in the CT images as well as demographic data.

Therefore, we propose a 10-point ordinal scoring system for IV-risk stratification, consisting of demographic data as well as imaging characteristics and distribution criteria that are all typically accessible from CT scan. Therefore, all data can reliably be determined by radiologists even in the emergency work-up under substantial time pressure. We accounted for the distribution of lesions, imaging features associated predominantly with later disease stages of the disease as well as CT findings reflecting possible cardiovascular comorbidities and systemic inflammation. For the demographic category, male gender was included as it turned out to be significantly different between the groups in our study cohort. Higher age is described as an important risk factor for disease severity and was therefore included in the score [36]. The three included distribution features reflect lung involvement as this was shown to be a decisive factor in previous studies [20,23,30]. All imaging characteristics which either turned out to be significantly different in group testing or showed an odds ratio of >2.0 for IV were selected. In order to avoid over-weighting of rare findings in an ordinary scoring system, only features with prevalence higher than 10% within the cohort were included. We aimed for a simple, clear and fast risk scoring model, using only data displayed to the radiologist during routine reporting. Our scoring system proved to accurately differentiate patients with high risk of invasive ventilation (IV) from patients with moderate disease courses (non-IV) with an AUC of 0.89 (95% CI 0.81–0.96). A cut-off value of 5 points was found to maximize sensitivity and specificity and thereby takes into account the scarce ICU capabilities during this crisis most accurately (sensitivity of 0.81 and specificity of 0.85). However, a cut-off of 4 points might adjust for a minimization of false negatively scored patients that will be in need of IV and thus should early on be monitored more closely and potentially be treated differently (sensitivity > 0.9, specificity > 0.65).

The advantage of our developed risk score lies in its simplicity and at the same time adequate discriminative ability, which facilitates the implementation into daily clinical practice. We believe that patients exhibiting a high IV-risk score result should be monitored closely for the need of invasive oxygen supply requirements and should possibly be transferred to centers of maximum care as their risk of developing a severe course is accordingly higher. The proposed score can feasibly be utilized during radiologic standard reporting and can be used as an imaging biomarker, which valuably contributes to the overall assessment of patient risk and therefore can facilitate decision making. As the numbers of COVID-19 infections still accelerate and continue to challenge health care systems globally during this ongoing pandemic, effective and resource-saving decision-making becomes all the more essential. Based on what has been shown and also on our experience in the first wave of the pandemic at our hospital, 15–30% of hospitalized SARS-CoV-2 patients may show critical courses of the disease being highly dependent on invasive ventilation [37,38], which in turn exerts high pressure on the health care system in general and critical care in particular. Therefore, early detection of patients who may require invasive oxygen supply is crucial in the process of optimizing available capacities in any health care system experiencing resource scarcity.

### Limitations

The proposed score derived from this study needs further investigation ideally on a larger validation cohort and over an extended validation period. One limitation of the study is its relatively low sample size due to a finite number of COVID-19 patients that could be treated in our hospital during the first wave of the pandemic. Furthermore, clinical information was limited to records in medical reports and no vital parameters were recorded for the study. Future research could focus on the additive value of additional clinical factors and laboratory parameters to be implemented into the score within in a multivariable model. Furthermore, we recommend that our image-based risk score is externally validated to verify the predictive performance regarding invasive ventilation therapy in an independent data set. In addition, it could be evaluated for its potential in a non-PCR confirmed patient cohort of COVID-19 patients.

## 5. Conclusions

The proposed invasive ventilation risk scoring model serves as a reliable and effective tool for risk stratification of COVID-19 patients in initial assessment upon hospital admission and supports effective allocation and resource management within hospitals and hospital networks.

## Figures and Tables

**Table 1:** Demographic data, clinical characteristics and epidemiologic features of our study cohort.

Data are *n* (%) if Not Indicated Differently	All Patients (*n* = 69)	Non-IV-Group (*n* = 46)	IV-Group (*n* = 23)	*p*-Values
Clinical and demographic data				
Age in years; median (95% CI)/ mean (SD)	62 (58.4–65.6)/ 61.0 (15.3)	61 (56.6–65.4)/ 61.5 (15.2)	64 (57.5–70.5)/ 60.2 (15.8)	0.944
Sex:				**<0.001**
*Male*	47 (68.0)	25 (54.0)	22 (96.0)	
*Female*	22 (32.0)	21 (46.0)	1 (4.0)	
Underlying disease:	57 (82.6)	36 (78.3)	21 (91.3)	0.985
*Cardiovascular*	40 (70.2)	23 (63.9)	17 (81.0)	
*Respiratory*	13 (22.8)	7 (19.4)	6 (28.6)	
*Immunologic/endocrinologic*	14 (24.6)	8 (22.2)	6 (28.6)	
*Obesity (BMI > 30)*	8 (14.0)	5 (13.9)	3 (14.3)	
No disease known:	12 (17.4)	10 (21.7)	2 (8.7)	0.178
**Laboratory data on admission** in mean (SD)				
CRP (mg/dL)	7.6 (8.2)	5.5 (6.1)	11.9 (9.9)	**0.004**
Procalcitonin (ng/mL)	1.0 (3.5)	0.2 (0.5)	2.4 (5.6)	**0.032**
Creatinine (mg/dL)	1.1 (0.8)	0.9 (0.5)	1.5 (1.0)	0.490
LDH (U/L)	374.0 (208.5)	326.2 (209.0)	469.6 (171.6)	**<0.001**
Leucocytes (×10^9^/L)	11.5 (36.5)	5.8 (2.9)	22.7 (61.0)	0.912
Lymphocytes (×10^9^/L)	1.1 (0.6)	1.1 (0.7)	1.0 (0.3)	0.849
IL-6 (ng/L)	884.6 (197.8)	34.1 (31.6)	482.3 (1351.2)	**<0.001**
INR	1.0 (0.1)	1.0 (0.2)	1.0 (0.1)	0.904
aPTT (seconds)	29.2 (4.6)	29.3 (4.9)	28.9 (4.0)	0.803
D-dimer (mg/L)	2.1 (4.5)	1.5 (1.8)	3.3 (7.1)	0.280
**Symptoms on admission**				
Dry cough	49 (71.0)	33 (71.7)	16 (69.6)	0.851
Fever	56 (81.2)	37 (80.4)	19 (82.6)	1
Sore throat/loss of taste	13 (18.8)	9 (19.6)	4 (17.4)	1
Reduced general condition	45 (65.2)	31 (67.4)	14 (60.9)	0.592
Limb pain/chills	13 (18.8)	8 (17.4)	5 (21.7)	0.663
Retrosternal/chest pain	11 (15.9)	9 (19.6)	2 (8.7)	0.314
Dyspnea	37 (53.6)	22 (47.8)	15 (65.2)	0.172
Diarrhea/nausea/vomiting	24 (34.8)	17 (37.0)	7 (30.4)	0.592
Dizziness/syncope/headache	10 (14.5)	6 (13.0)	4 (17.4)	0.721
**Symptoms onset**				
Week of symptoms; mean (SD)	1.9 (0.75)	1.7 (0.73)	2.4 (0.75)	**0.010**
**Outcome parameters**				
Length of hospital stay (days)	21.0 (32.4)	9.7 (7.6)	43.6 (47.7)	**<0.001**
In-hospital mortality	7 (10.1)	2 (4.3)	5 (21.7)	**0.039**
Time from admission to intubation (days)	-	-	2.9 (3.1)	

IV = Invasive ventilation group; non-IV = No/non-invasive ventilation group; significant results are highlighted in bold; subcategories are italicized; cardiovascular diseases: arterial hypertension, pulmonary hypertension, coronary heart disease, aortal aneurysm, valvular hearth disease, cardiomyopathies, cardiac arrhythmia, hypercholesterinemia; pulmonary diseases: chronic obstructive pulmonary disease, recurrent pneumonia, interstitial lung diseases, nicotine abuse; immunologic/endocrinologic diseases: diabetes mellitus, immune suppression (HIV, chemotherapy).

**Table 2:** Computed tomography (CT) and chest radiograph (CXR) imaging features of our study cohort.

CT Features Data are *n* (%) if Not Indicated Differently	All Patients (*n* = 69)	No/Non-Invasive Ventilation Group (*n* = 46)	Invasive Ventilation Group (*n* = 23)	*p*-Values	Odds Ratios (OR)
Native high-resolution (HR-CT) protocol	53 (76.8)	39 (84.8)	14 (60.9)		
Contrast-enhanced (CE) pulmonary embolism protocol:	16 (23.2)	7 (15.2)	9 (39.1)		
*Pulmonary embolism present*	3 (18.6)	2 (28.6)	1 (11.1)	0.550	
**Imaging findings**					
Ground glass opacities (GGOs)	63 (91.3)	40 (87.0)	23 (100.0)	0.168	-*
Consolidations	37 (53.6)	25 (54.3)	12 (52.2)	0.864	0.91
Crazy paving	22 (31.9)	14 (30.4)	8 (34.8)	0.715	1.11
Subpleural bands	24 (34.8)	12 (26.1)	12 (52.2)	**0.032**	**3.09**
Air bronchogram	32 (46.4)	20 (43.5)	12 (52.2)	0.495	**2.60**
Architectural distortion/emphysema	16 (23.2)	13 (28.3)	3 (13.0)	0.158	1.24
Bronchial wall thickening/mucus-plugging	5 (7.2)	4 (8.7)	1 (4.3)	0.511	0.48
Reticulations	9 (13.0)	5 (10.9)	4 (17.4)	0.448	1.73
Lymph node enlargement	8 (11.6)	7 (15.2)	10 (43.5)	**0.010**	**4.30**
Pulmonary hypertension	11 (15.9)	5 (10.9)	6 (26.1)	0.104	1.59
Cardiomegaly	7 (10.1)	2 (4.3)	5 (21.7)	**0.037**	**6.92**
Coronary artery disease	27 (39.1)	15 (32.6)	12 (52.2)	0.116	**2.27**
Pericardial effusion	3 (4.3)	2 (4.3)	1 (4.3)	1.000	1.0
Pleural effusion	5 (7.2)	1 (2.2)	4 (17.4)	**0.039**	**9.55**
**Distribution**					
Number of lobes; mean (SD)	**4.04 (1.7)**	**3.5 (1.9)**	**4.9 (0.6)**	**0.008**	
Regional distribution:					
*Bilateral*	56 (81.2)	34 (74.0)	22 (95.7)	0.241	
*Unilateral*	8 (11.6)	7 (15.2)	1 (4.3)		
Craniocaudal distribution:					
*Upper lobe predominance*	7 (10.1)	6 (13.0)	1 (4.3)	**0.004**	
*Lower lobe predominance*	30 (43.5)	24 (52.2)	6 (26.1)		
*Both upper and lower lobes*	27 (39.1)	11 (24.0)	16 (69.6)		
Transverse distribution:					
*Peripheral predominance*	49 (71.0)	35 (76.1)	14 (60.9)	0.055	
*Central predominance*	2 (2.9)	1 (2.2)	1 (4.3)		
*Both central and peripheral*	13 (18.8)	5 (10.9)	8 (34.8)		
Spread:					
*Focal*	6 (8.7)	5 (10.9)	1 (4.3)	**<0.001**	
*Multifocal*	42 (60.9)	33 (71.7)	8 (34.8)		
*Diffuse*	17 (24.6)	3 (6.5)	14 (60.9)		
No CT findings	5 (7.2)	5 (10.9)	0 (0.0)		
CT lung involvement score (0-20); mean (SD)	**13.01 (6.5)**	**10.76 (6.6)**	**17.52 (3.0)**	**<0.001**	
CXR present 48 h < or > of CT scan	31 (44.9)	11 (23.9)	20 (87.0)		
Indicative signs for pulmonary involvement	27 (87.1)	7 (63.6)	20 (100.0)	**0.010**	

IV = Invasive ventilation group; non-IV = No/non-invasive ventilation group; significant results are highlighted in bold; subcategories are italicized; * not available due to 100% presence of GGOs in IV-group.
